# Special Issue: Antibacterial Materials and Coatings

**DOI:** 10.3390/molecules23030585

**Published:** 2018-03-06

**Authors:** Krasimir Vasilev, Alex Cavallaro, Peter Zilm

**Affiliations:** 1 School of Engineering, University of South Australia, Adelaide, SA 5095, Australia; 2 Future Industries Institute, University of South Australia, Adelaide, SA 5095, Australia; alex-anthony.cavallaro@unisa.edu.au; 3 Adelaide Dental School, The University of Adelaide, Adelaide, SA 5005, Australia; peter.zilm@adelaide.edu.au

The problems associated with undesired bacterial adhesion and colonization of surfaces are highly recognized globally and are well documented. Biofilm formation directly affects the performance of many industrial processes, such as food processing and storage, marine transport and management, and water treatment. In the medical field, bacterial colonization of medical devices, including surgical equipment, implants and other healthcare related products, poses serious risks to public health. Such biofilms and infections are generally combated with a variety of antimicrobial agents; however, the ability of microorganisms to develop resistance or complete immunity to antimicrobial compounds, including many antibiotics, has made microbiological control increasingly difficult. Despite the continued research efforts towards prevention of biofouling and additional controls implemented by industry, a comprehensive solution to microbial surface colonization is yet to be found.

This Special Issue of Molecules is dedicated to presenting the latest advances in the field of antibacterial materials and coatings. The Special Issue consists of one review and 24 original research articles, which in our opinion make substantial and interesting contributions to the field.

Owing to its high biocompatibility, low toxicity and antimicrobial properties, the use of chitosan in antimicrobial coatings and materials is a rapidly growing research area. In this Special Issue, four articles highlight the use of chitosan as an antibacterial agent or coating and evaluate its potential. Ardila et al. (Antibacterial Activity of Neat Chitosan Powder and Flakes) thoroughly investigated the antimicrobial capacity of different forms of chitosan against bacteria which frequently cause food spoilage. The influence on environmental factors such as temperature range, pH and ionic strength on chitosan efficacy were all shown [1]. Arkoun et al. reported on the “Mechanism of Action of Electrospun Chitosan-Based Nanofibers against Meat Spoilage and Pathogenic Bacteria” discussing the susceptibility of *E. coli*, *L. innocua*, *S. aureus* and *S. typhimurium* to chitosan-based nanofibers [2]. Kulig et al. (Effect of Film-Forming Alginate/Chitosan Polyelectrolyte Complex on the Storage Quality of Pork) developed sodium alginate-chitosan polyelectrolyte complexes, which significantly reduced lactic acid bacteria by around 61%, and molds by about 45%, after 2 weeks [3]. These findings point towards the great potential that chitosan-based materials have in food processing and storage. However, the use of chitosan’s antibacterial properties is not limited to food-based applications. Chitosan and polyhaxametyle guanidine dual-polymer functionalized graphene oxide were shown to be an effective antibacterial agent by Li et al. These novel graphene oxides exhibit extremely high antibacterial activity against both Gram-positive and Gram-negative bacteria. In vitro evaluation of these dual-polymer graphene oxides revealed minimum inhibitory concentrations of 32 µg/mL against medically relevant *E. coli* [4].

Another highly popular research direction towards effective antibacterial coatings involves the use of sliver. Silver-based coatings and silver nanoparticles (AgNP) are becoming an increasingly common as antibacterial agents and are commercially employed within the medical field. Cai et al. developed a one-step process to synthesize AgNP on polydopamine-coated sericin/polyvinyl alcohol films without the use of reducing agents. These films showed good long-term stability and antibacterial activity against both Gram-positive and Gram-negative bacteria [5]. In another excellent research article, Jankauskaite et al. (UV-Curable Aliphatic Silicone Acrylate Organic–Inorganic Hybrid Coatings with Antibacterial Activity) present a novel process for the synthesis of organic–inorganic hybrid coatings containing silver nanoparticles. Due to the release of silver ions from the coatings, these coatings are highly effective antimicrobial coatings [6]. Ziabka et al. prepared a middle ear prosthesis consisting of ABS and AgNP. The ABS-AgNP composites were proven to release silver ions at a concentration capable of exhibiting great antimicrobial effect but remain biocompatible [7]. In another application-based research article, AgNPs were synthesized in situ on an orthodontic elastomeric modules material using silver nitrate salts and Hetheroteca inuloides extracts as an eco-friendly bioreductant. Here, Hernández-Gómora et al. assessed the efficacy of these materials against clinical isolates of S. *mutans*, *L. casei*, *S. aureus* and *E. coli*. The promising results suggest that these materials could be applied to combat dental biofilms and ensure the performance of the orthodontic materials in clinical use [8].

The use of magnetron sputtered metals for the generation of antimicrobial surfaces is also presented in this special issue. Goderecci et al. (Silver Oxide Coatings with High Silver-Ion Elution Rates and Characterization of Bactericidal Activity) utilized reactive magnetron sputtering to synthesize AgO, Ag_2_O and mixtures of AgO, Ag_2_O. These coatings elute silver ions to concentrations to bactericidal towards *S. aureus* and *E. coli*. The elution of Ag^+^ was also shown to have no effect on cell mammalian apoptosis after 24 h [9]. In an excellent review paper, Jindrich Musil explains the use of reactive magnetron sputtering to produce coatings that are not only antimicrobial but have the capacity to be deposited directly onto flexible substrates. Insights into how chemical compositions of coatings of Cr–Cu–O, Al–Cu–N or Zr–Cu–N can be tailored to produce films with greater antibacterial properties [10].

Valdez-Salas et al. prepared of micro/nanostructured metal surfaces and discussed the early bactericidal activity of smooth, rough and nanostructured surfaces. It was revealed that the nano-rough Ti6Al4V surfaces decreased adhesion and viability of *S. aureus* and *P. aeruginosa* [11]. The antibacterial efficacy of Ti_6_A_l4_V substrates coated with a hydroxyapatite-AgNP composite was evaluated by Lozoya-Rodriuez et al. It was shown that these surfaces exhibit 99.99% efficacy against *S. aureus*, *E. coli* and *P. aeruginosa* [12]. An in vivo study of Cu-TiO_2_ as an antimicrobial coating is also presented in this Special Issue. Mauerer et al. revealed that these coating impart good antimicrobial properties without impairment of liver and kidney function [13].

Iconaru et al. (Structural Characterization and Antifungal Studies of Zinc-Doped Hydroxyapatite Coatings) developed zinc-doped hydroxyapatite coatings and evaluated their antifungal potential against *Candida albicans*. These coatings showed no activity when incubated in the dark. However, upon activation of the surface under daylight and UV light illumination, they were able to significantly reduce the viability of *C. Albicans* [14]. Similarly, Mizielinska et al. showed that methyl-hydroxypropyl cellulose doped with ZnO nanoparticles was highly effective as an antimicrobial under similar conditions [15].

Other approaches towards antibacterial surfaces, such as the use of phenolic compounds against food and beverage spoiling [16,17], the rational design and evaluation of antimicrobial properties of phenyl-diazenyl(phenols), Gemini-surfactants, novel azo-compounds and N-Halamine materials [18,19,20,21] and use of essential oils [22,23] are also presented in this Special Issue. This Special Issue also reports on the regulation of dental microecosystems, using implants coated with Dimethylaminododecyl Methacrylate [24] or the introduction of new oral probiotics [25].

Finally, the Editorial team would like to thank all contributing authors for making this Special Issue a success.
